# Analyzing Surface Roughness Variations in Material Extrusion Additive Manufacturing of Nylon Carbon Fiber Composites

**DOI:** 10.3390/polym15173633

**Published:** 2023-09-01

**Authors:** Muhammad Abas, Mohammed Al Awadh, Tufail Habib, Sahar Noor

**Affiliations:** 1 Department of Industrial Engineering, University of Engineering & Technology, Peshawar 25100, Pakistan; tufailh@uetpeshawar.edu.pk (T.H.); sahar@uetpeshawar.edu.pk (S.N.); 2 Department of Industrial Engineering, King Khalid University, Farah 64231, Saudi Arabia

**Keywords:** fused deposition modeling, surface roughness, nylon carbon fiber, definitive screening design, composite desirability function, entropy

## Abstract

In recent years, fused deposition modeling (FDM) based on material extrusion additive manufacturing technology has become widely accepted as a cost-effective method for fabricating engineering components with net-shapes. However, the limited exploration of the influence of FDM process parameters on surface roughness parameters, i.e., Ra (average surface roughness), Rq (root mean square surface roughness), and Rz (maximum height of the profile) across different sides (bottom, top, and walls) poses a challenge for the fabrication of functional parts. This research aims to bridge the knowledge gap by analyzing surface roughness under various process parameters and optimizing it for nylon carbon fiber printed parts. A definitive screening design (DSD) was employed for experimental runs. The Pareto chart highlighted the significant effects of layer height, part orientation, and infill density on all surface roughness parameters and respective sides. The surface morphology was analyzed through optical microscopy. Multi-response optimization was performed using an integrated approach of composited desirability function and entropy. The findings of the present study hold significant industrial applications, enhancing the quality and performance of 3D printed parts. From intricate prototypes to durable automotive components, the optimized surfaces contribute to production of functional and visually appealing products across various sectors.

## 1. Introduction

Additive manufacturing (AM) is a transformative manufacturing process that builds objects layer by layer using digital computer-aided design data. It offers several advantages over traditional manufacturing methods. AM allows for creating complex geometries that are challenging or impossible to manufacture with conventional techniques. It offers design freedom, enabling customization, rapid prototyping, and on-demand production. It facilities reduced material waste, lower tooling costs, and shorter lead times. AM is poised to revolutionize the manufacturing landscape as technology continues to evolve, enabling innovation and opening up new possibilities in product development and production [1,2,3].

Fused deposition modeling (FDM) among AM technologies is a popular choice in the industry due to its affordability, ease of use, material options, and broad application. However, achieving a smooth surface finish with FDM may have limitations compared to other AM technologies like SLA (Stereolithography) or SLS (Selective Laser Sintering). These technologies often produce smoother surfaces due to their different printing processes and finer resolutions [4,5,6,7]. In FDM printing, surface roughness is influenced by various factors, including process parameters, material properties, and geometric features. To optimize surface roughness in FDM printing, different systematic approaches have been proposed based on statistical methods (namely an experimental design and analysis of variance (ANOVA)), evolutionary algorithms, and artificial neural networks (ANN). This involves adjusting process parameters, such as printing speed, nozzle temperature, layer height, raster angle, bed temperature, nozzle size, and infill density, etc., to find the optimal combination that balances surface quality with other performance requirements. Additionally, selecting filaments with smoother characteristics or exploring specialized coatings designed for FDM parts can further enhance the surface finish.

The most common surface roughness parameters are Ra (average roughness), Rq (root mean square roughness), and Rz (maximum height of the profile). Ra, Rq, and Rz are commonly used surface roughness parameters in additive manufacturing (AM) because they provide valuable insights into the quality and characteristics of the printed surfaces. They are internationally recognized parameters standardized in various surface roughness standards, such as ISO 21920 [8] and ANSI/ASME B46.1 [9]. This standardization facilitates consistent and reliable measurement and comparison of surface roughness values across different AM processes and materials. Ra, Rq, and Rz correlate reasonably well with friction, wear, adhesion, and contact mechanics [10,11]. By quantifying the surface roughness using these parameters, designers and engineers can assess how the printed part’s surface quality will affect its functionality and performance in specific applications [11]. Ra, Rq, and Rz are relatively simple to measure using profilometric or surface roughness measurement devices. The measurement techniques are widely available and easily implemented, making these parameters practical for routine quality control and inspection in AM processes [12].

The main objective of the present study is four-fold. Firstly, it aims to investigate and comprehend the impact of fused deposition modeling (FDM) process parameters on surface roughness parameters (Ra, Rq, and Rz) across different sides (bottom, top, and walls) of nylon carbon fiber printed components. Secondly, an experimental design approach utilizing a definitive screening design (DSD) with three levels is employed for experimentation, with a specific focus on the printing process parameters. Thirdly, a comprehensive statistical analysis is performed to assess the effect of various printing process parameters on the surface roughness parameters. Additionally, surface morphology is analyzed through the use of optical microscopy. Lastly, the study conducts multi-response optimization by integrating composited desirability function and entropy methods. The ultimate goal is to significantly enhance the understanding of surface roughness and its optimization for functional parts manufactured using FDM technology.

## 2. Literature Review 

The surface roughness of FDM printed parts has been studied in several studies and has been optimized for different materials based on different process parameters. Mushtaq et al. [13] conducted an optimization study on the average surface roughness (Ra) and root mean square roughness (Rq) of acrylonitrile butadiene styrene (ABS) and Nylon-6 (PA-6) and printed parts. They employed Taguchi S/N ratios, an ANOVA, and regression modeling techniques. The optimized settings resulted in a surface roughness of 1.75 µm for ABS and 21.37 µm for PA-6. The significant parameters identified for minimizing surface roughness in ABS were the initial layer height, width of the raster, and temperature of the bed, while for PA-6, they were the layer thickness, print speed, and extrusion temperature. Chhabra et al. [14] optimized Ra for nylon carbon fiber composite parts based on an integrated approach of an artificial neural network and genetic algorithm (ANNGA). The ANNGA showed promising results for prediction, optimization, and improvement in various engineering applications. Using response surface methodology (RSM) and a multi-objective-genetic-algorithm–artificial-neural-network (MOGA-ANN) approach, Gahletia et al. [15] optimized the Ra, tensile strength, and wear rate of nylon carbon fiber composite parts. The MOGA-ANN approach demonstrated superior results by maximizing tensile strength while minimizing the wear rate and Ra. In a study by Rashed et al. [16], FDM process parameters were compared with Taguchi and full factorial designs of experiments (DoEs) to assess the effects on mechanical and surface roughness properties of Nylon 6/66. The findings revealed that mechanical and surface properties are influenced by infill density, while layer height primarily affected the manufacturing time for the specific polymer. The Taguchi DoE approach demonstrated its efficiency in determining optimal process parameters, particularly for linear responses.

Mishra et al. [17] optimized the dimensional errors and surface finish of polyethylene terephthalate glycol (PETG) parts. The layer height and raster width were found to be significant. Further, ANFIS models were also developed, outperforming RSM with a low root mean square error. RSM combined with the NSGA-II algorithm was employed to identify optimized parameters. For ABS printed parts, Vyavahare et al. [18] performed a comprehensive investigation on the effect of FDM process parameters on Ra, printing time, and dimensional accuracy. The layer height, orientation of the part, and printing speed were found to be significant for all considered responses. The regression model developed was non-linear quadratic and able to predict these responses accurately. Further, desirability function was performed to achieve optimal results. Saad et al. [19] utilized RSM along with symbiotic organism search (SOS) and particle swarm optimization (PSO) techniques to minimize Ra of PLA printed parts. The layer height, followed by the printing speed, extrusion temperature, and printing speed of outer shells, were found to be significant. The results showed that SOS and PSO algorithms performed better than the RSM alone.

Nagendra et al. [20] optimized Ra for nylon and Aramid printed parts. They employed a Taguchi experimental design approach. The findings indicated that optimal surface smoothness was achieved. The validated part demonstrated a combined objective error of 2.45%, attributed to potential hardware or operating condition variations. The results of Chand et al. [21] showed that dimensional accuracy and surface roughness were significantly altered by changing the print orientation of the part. The most suitable orientation for achieving the desired surface quality and dimensional accuracy is the part lying on the base. According to Chohan et al. [22], the Ra was predominantly impacted by printing speed, while nozzle temperature had a minor effect in ABS parts. In their study, Chinchanikar et al. [23] developed an ANN model for Ra prediction of PLA parts. The best prediction accuracy was obtained and validated. The study found that increasing infill density reduces surface roughness, while increasing layer thickness, printing speed, and extrusion temperature causes an increase in surface roughness. Buj-Corral et al. [24] concluded that dimensional error and surface roughness are significantly influenced by the layer height and flow rate, while impact porosity is only influenced by the layer height. Cerro et al. [25] employed machine learning algorithms to predict Ra of polyvinyl butyral printed parts. The best-performing model was developed using bagging and Multilayer Perceptron (BMLP). The layer height and wall angle were found to be significant for Ra. Venkatraman and Raghuraman [26] performed a parametric analysis of ABS printed parts using a Box–Behnken design (BBD). The study found that the percentage of infill, layer thickness, and temperature of the bed significantly impacted the processing time, part density, micro-hardness, and surface roughness. Nugroho et al. [27] indicated that layer height significantly affects printed samples’ surface finish and dimensional accuracy. Kholil et al. [28] demonstrated that an increase in the orientation angle and layer height causes an increase in surface roughness. Higher surface roughness was observed at an orientation angle of 60° and a layer thickness of 0.15 mm. According to a study of Khunt et al. [29], higher printing temperature and lower printing speed and layer thickness were found to improve the surface finish and density of polyetheretherketone (PEEK) parts. The surface roughness of PETG printed parts was optimized by Ulkir and Akgun [30] using a combined approach of a conventional feedforward neural network (CFNN) and a genetic algorithm (GA). Their research demonstrated the hybrid CFNN-GA approach, in combination with a Box–Behken design (BBD), and effectively predicted and minimized the surface roughness, achieving error rates below 10%. Additionally, the study revealed that thinner layers contributed to reduced roughness, while an increased wall thickness and nozzle temperature resulted in higher roughness levels. A combined approach of an artificial neural network (ANN) and whale optimization algorithm (WOA) was used by Kumar et al. [31] to improve the surface roughness of PLA printed parts. Results showed that the model performed better in terms of prediction. According to their study, nozzle temperature and layer thickness have a significant impact on surface roughness. Increasing layer thickness increased roughness, while reducing layer thickness reduced roughness.

The literature review identified that minimizing the surface roughness in FDM printed parts is challenging. This difficulty arises primarily due to its dependency on process parameters, the material type, and the anisotropic nature of printed parts. It is challenging to determine the optimal combination of process parameters for different types of materials, since each material has its own flow characteristics, cooling rates, and adhesion properties, all of which have an effect on surface roughness and require process adjustments. FDM printed parts exhibit anisotropic behavior, meaning their mechanical properties and surface quality can vary depending on the printing direction. A limited amount of literature is available regarding the effect of FDM process parameters on the surface roughness parameters, namely Ra (average surface roughness), Rq (root mean square surface roughness), and Rz (maximum height of the profile) of nylon carbon fiber printed parts, as it is one of the challenging materials to print through due to its unique characteristics. Optimizing print settings based on filament manufacturer guidelines, an experimental design, and artificial intelligence (AI) based on nature-inspired metaheuristic approaches is vital to address these challenges. There is a lack of studies that specifically investigate the impact of FDM process parameters on the surface quality of different sides of printed parts, such as the bottom, top, and walls. The top surface is directly exposed to the environment and is the last layer printed in the vertical direction. The bottom surface is typically in contact with the build platform and is the foundation for the rest of the print. Walls refer to the vertical sides of a printed object. Additionally, in the literature, the combination of FDM process parameters such as the layer height, print speed, infill density, part orientation, number of shells, fill angle, extrusion temperature, and bed temperature are given limited attention for a nylon carbon fiber composite, so it suggests potential research opportunities to explore the synergistic effects of these parameters on surface roughness parameters of different-side printed parts. Further investigations in this area can contribute to a deeper understanding of process optimization for printing with nylon carbon fiber composites. Finally, an integrated approach of composite desirability function (CDF) and entropy is employed for multi-response optimization. In a nutshell, this study can contribute to the existing body of knowledge and help bridge the current gaps in understanding the influence of FDM process parameters on surface roughness for nylon carbon fiber printed parts.

## 3. Materials and Methods

In the present study, the filament used for printing is nylon carbon fiber supplied by Machines-3D^®^, Valenciennes, Nord-Pas-de-Calais, France. According to manufacturer specifications, it is derived from a monomer, polyamide 12 (PA12), with the addition of a 20% carbon fiber component. The diameter of the filament is 1.75 mm. It has a heat distortion/deflection temperature of 155 °C, a 1.00 g/cm^3^ density, and a melting temperature of 180 °C. It is an engineering polymer composite that offers an excellent balance of strength, stiffness, and heat resistance, making it suitable for various industrial applications. It is commonly used in aerospace, automotive, robotics, and prototyping industries, requiring parts with high mechanical performance and durability [32,33].

**Figure 1:**
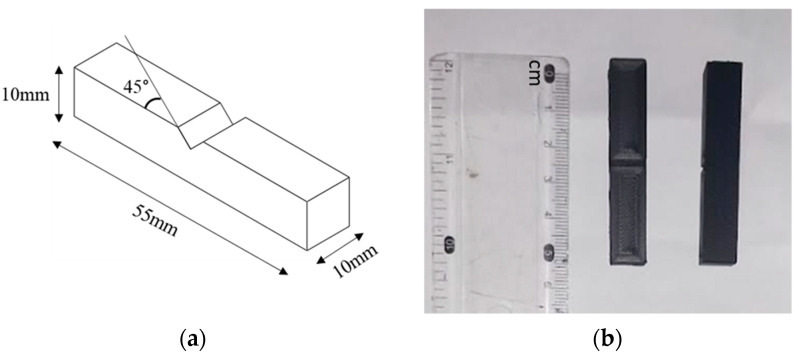
(**a**) Schematic of test specimen. (**b**) FDM printed specimen from nylon carbon fiber.

The parts were printed using a Voron Trident 3D (Voron Design, Guangdong, China) printer and are commonly used for impact testing, according to ASTM E23-23a [34], as shown in Figure 1. Each test sample was fabricated on the printer bed individually to have similar values and properties as recommended in previous studies [35,36].

Process parameters considered were the layer height, print speed, infill density, part orientation, number of shells, fill angle, extrusion temperature, and bed temperature. Three levels were set for each process parameter based on the recommendation of the material manufacturer and literature review [13,37,38,39], as presented in Table 1.

### 3.1. Experimental Design

The experiments employ the three-level definitive screening design (DSD) approach. The DSD, pioneered by Jones and Nachtsheim [40], is a powerful approach that combines screening, response surface investigation, and optimization in a single experimental design. This design stands out from traditional screening designs by providing estimates not only for main effects but also for interaction and quadratic effects, all while using a relatively small number of experimental runs. Compared to traditional screening designs, which often require a more significant number of experimental runs, the three-level DSD offers exceptional efficiency. With only one additional run, the number of experimental runs is just twice that of factors. Reducing the number of runs makes the design particularly advantageous when the resources are limited. An essential feature of this design is its ability to estimate the main effects independently of the interaction and quadratic effects. This independence allows for a clear understanding of the impact of each factor on the response variable without confounding effects from other interactions or quadratic terms. Furthermore, the design’s two-factor interaction and quadratic effects are not entirely confounded with each other or with other two-factor interactions. This means that the design allows for the separate estimation of these effects, enabling a comprehensive analysis of the system under investigation. The three-level definitive screening design streamlines the experimental process by incorporating screening and response surface investigation into a single design. It efficiently identifies significant factors, quantifies their effects, and explores potential interactions and quadratic relationships. This valuable information serves as a solid foundation for subsequent optimization efforts.

So, in the present study, considering eight process parameters at three levels, a total of 17 experimental runs were planned based on DSD. However, recognizing the significance of repeatability and robustness, we took an extra step by randomly repeating each experiment three times. By incorporating these repetitions, the inherent variability of the printing process can be captured efficiently, and the results can be more reliable and precise. The final design, therefore, encompasses a total of 51 experimental runs, as tabulated in Table 2, providing a comprehensive dataset for an analysis. It enables us to gain deeper insights into the effects of the process parameters and their interactions and optimize the printing process more effectively.

### 3.2. Surface Roughness Measurement

**Figure 2:**
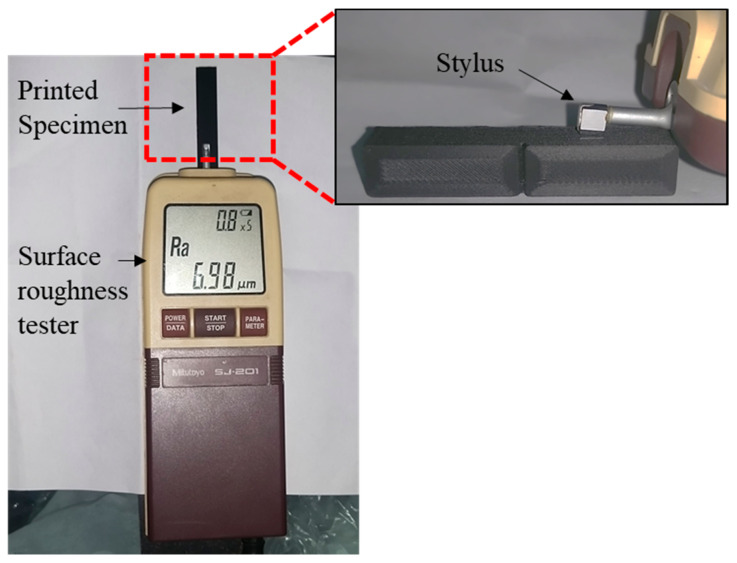
Surface roughness measurement of a printed specimen using a Mitutoyo surface roughness tester.

A Mitutoyo surface roughness tester (SJ-201, Mitutoyo Corporation, Kanagawa, Japan) was used to measure Ra, Rz, and Rq using the stylus method, as shown in Figure 2. The measurement procedure followed the guidelines outlined in the ISO 21920 [8] industrial standard. Five readings perpendicular to the printed payers of each specimen were taken to ensure reliable and representative measurements. This approach helps account for any variations in surface roughness within the printed layers. The Mitutoyo surface roughness tester had specific settings, including an evaluation length of 4 mm, a tip angle of 90°, a tip radius of 2 µm, a tracing speed of 0.25 mm/s, and a cut-off wavelength of 0.8 mm. These settings were standardized to maintain consistency and accuracy across all measurements. Finally, based on collected measurements, the average values of Ra, Rz, and Rq were computed for each specimen from the top, wall, and bottom surfaces. The resulting average values are presented in Table 3, providing an overview of the surface roughness characteristics of the printed specimens.

### 3.3. Methodology for Multi-Response Optimization

#### 3.3.1. Composite Desirability Function

Derringer [41] developed the composite desirability function for multi-response optimization. The idea behind this approach is to convert estimated response models into individual desirability functions (*d*) and to aggregate them into a composite desirability function (*D*). Depending upon the objective function, it can be divided into three main categories: larger-the-best, nominal-the-best, and smaller-the-best. As the present study focuses on minimizing the surface roughness, the smaller-the-best objective function was used, as expressed in Equation (1) [41].

(1) $$d_{i}=\left[\begin{array}{c} \;\;\;\;\;\;\;\;0,\;\;\;\;\;\;\;\;\;\;\;\;\;\;\;\;\;\;\;\;\;\;\;\;\;\;\;\;\;\;\;\;\;\;\;\;y_{i}\;\leq \;\;\;\min\left(y_{i}\right)\;\;\;\;\;\;\;\;\;,\\ {\left[\frac{y_{i}-\max(y_{i})}{\min(y_{i})-\max(y_{i})}\right]}^{g}\;\;\;\;\;\;\min\left(y_{i}\right)\;\;\leq y_{i}\;\;\leq \;\max\left(y_{i}\right)\;\\ 1,\;\;\;\;\;\;\;\;\;\;\;\;\;\;\;\;\;\;\;\;\;\;\;\;\;\;\;\;\;\;\;\;\;\;\;\;\;\;\;y_{i}\;\geq \;\;\;\min\left(y_{i}\right) \end{array}\right]\;\;\;$$

where $d_{i}$ is the desirability value of the response at experiment i. $y_{i}$ is the measured response at experiment i. Max $y_{i}$ and min $y_{i}$ are the maximum and minimum. g is the weight that governs the desirability function distribution on the interval 0.1 to 10. For g < 1, it shows that lower importance is assigned to the target, and the desirability function becomes exponential (concave down and increasing). For g = 1, the desirability function becomes linear and equal importance is assigned to upper and lower limits and targets. For g > 1, the desirability function becomes exponential (concave up), and it demonstrates that more importance is assigned to the target, and the response moves closer to the desired target value.

The desirability values fluctuate between 0 and 1. If the desirability value for a particular experimental run becomes equal to or near 0, then it shows that the desired experimental run is unacceptable. If the desirability value for a specific experimental run becomes equal to or near one, it shows that the expected experimental run is acceptable and that the experimental run will be considered optimal with that highest desirability value.

Further, the desirability functions (*d*) are combined into a composite desirability function (*D*) using the geometric mean for multi-response optimization using Equation (2) [41].

(2) $$D_{i}=\prod_{j=1}^{r}{d_{j}}^{w_{j}}\;$$

where $w_{j}$ is the individual weight of response j.

CDF was used because it allows for incorporating multiple, potentially conflicting objectives and constraints into the optimization process consistently and intuitively and is recommended for second-order optimization problems based on response surface methodology (RSM) [42].

#### 3.3.2. Entropy

The entropy method was employed to calculate the criteria weights due to its broad applicability in real-time data analyses. It is known for effectively handling diverse datasets and providing reliable weight allocation results [43].

The following steps were deployed to calculate the weights of responses based on entropy.

Step 1: Compute the entropy of each desirability function of the response using Equations (3)–(5) [43].

(3) $$e_{j}=-k\sum_{i=1}^{m}\left(\mu_{ij}\times \ln\left(\mu_{ij}\right)\right)$$

(4) $$k=\frac{1}{ln(m)}$$

(5) $$\mu_{ij}=\frac{x_{ij}}{\sum_{i=1}^{m}x_{ij}}$$

where $\mu_{ij}$ is the normalized value, $x_{ij}$ is the original value in the decision matrix, and m is the number of experiments.

Step 2: Determine the redundancy of entropy (degree of diversity) using Equation (6) [43].

(6) $$d_{j}=1-e_{j}\;$$

Step 3: Finally, compute the weight of the response using Equation (7) [43].

(7) $$w_{j}=\frac{d_{j}}{\sum_{j=1}^{n}d_{j}}$$

**Figure 3:**
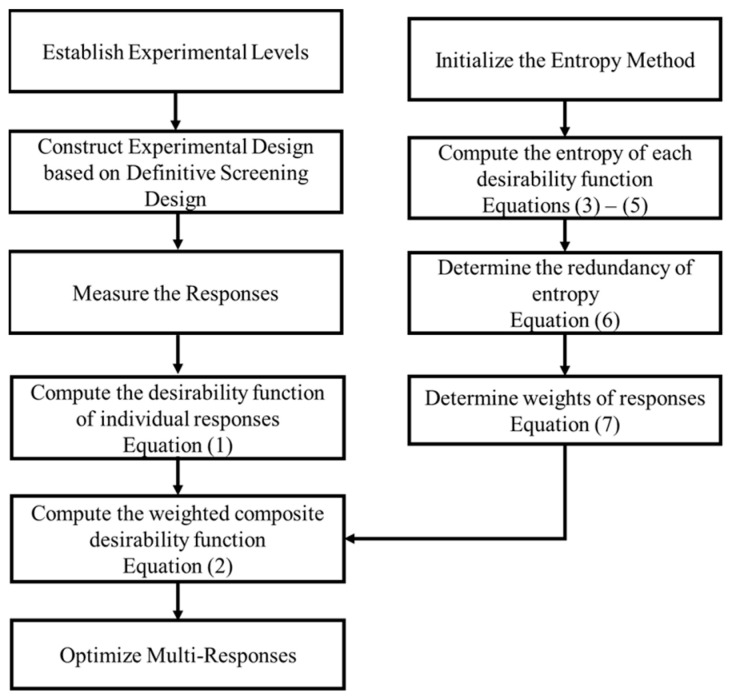
Methodology for multi-response optimization.

Figure 3 summarizes the methodology adopted for multi-response optimization.

## 4. Results and Discussion

### 4.1. Analysis of Variance (ANOVA)

**Figure 4:**
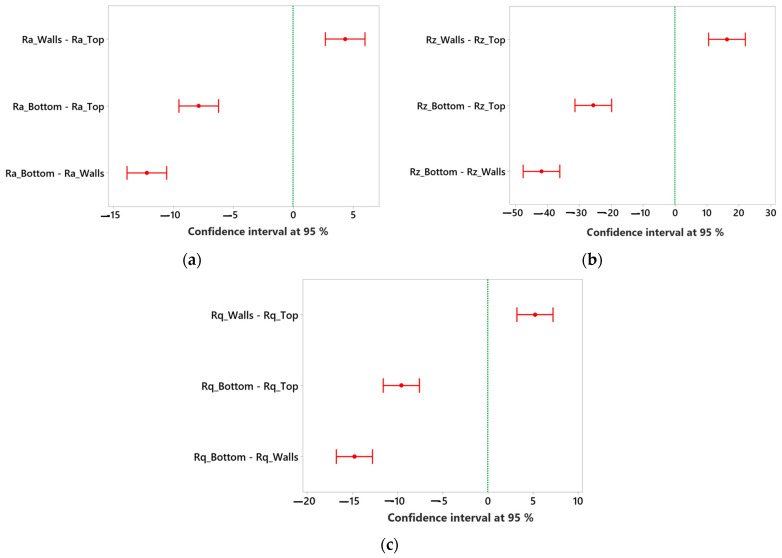
Tukey test for difference of means. (**a**) Ra for top, walls, and bottom surface. (**b**) Rz for top, walls, and bottom surface. (**c**) Rq for top, walls, and bottom surface.

Table 3 shows that the Ra, Rq, and Rz of walls are relatively higher than the top surface while lowest for the bottom surface. So, a statistical analysis based on ANOVA was conducted at a 95% confidence interval to determine if there are significant differences in the mean values of surface roughness parameters for the top, wall, and bottom surfaces of printed parts. Two hypotheses were set, including null and alternative hypotheses, at a significance level/alpha value of 0.05. The null hypothesis assumes that all means are equal (if the *p*-value of the test is greater than the alpha value of 0.05). In contrast, the alternative hypothesis states that not all means are equal or that there is a difference in at least one group mean (if the *p*-value of the test is less than or equal to the alpha value of 0.05). This analysis allows for a comprehensive assessment of the differences in mean values, helping to identify any significant variations in surface roughness parameters between the top, wall, and bottom sides. The results are summarized in Table 4. It shows that the *p*-values of the test are less than the alpha value of 0.05, depicting that at least two group means are significantly different. So, for this purpose, a statistical test based on the Tukey Simultaneous test is performed. In Figure 4, if an interval does not contain zero, the corresponding means are significantly different. It shows that all the groups are away from the reference line and are found to be significant, i.e., the mean values of Ra, Rz, and Rq for walls vary significantly from the mean values at the top and bottom surface. Similarly, mean values of Ra, Rz, and Rq of the top surface vary considerably from the bottom surfaces.

In Figure 4a, Ra_Walls exhibits a positive mean difference (4.33) over Ra_Top, suggesting a higher mean value for Ra_Walls. The 95% confidence interval (2.68, 5.98) adds confidence in the likely range of this difference. Conversely, Ra_Bottom shows a negative mean difference (−7.89) compared to Ra_Top, implying a lower average value for Ra_Bottom. The 95% confidence interval (−9.54, −6.24) reinforces this observation. Similarly, Ra_Bottom has a negative mean difference (−12.22) against Ra_Walls, pointing to a lower average value for Ra_Bottom. The 95% confidence interval (−13.87, −10.57) supports this finding.

In Figure 4b, Rz_Walls shows a positive mean difference (16.22) over Rz_Top, indicating a higher mean value for Rz_Walls. The relatively wide 95% confidence interval (10.47, 21.97) emphasizes this difference with reasonable certainty. On the other hand, Rz_Bottom displays a substantial negative mean difference (−25.64) compared to Rz_Top, suggesting a significantly lower mean value for Rz_Bottom. The 95% confidence interval (−31.39, −19.89) highlights the likely range of this difference. Moreover, Rz_Bottom exhibits a negative mean difference (−41.86) against Rz_Walls, reinforcing its lower average value. The 95% confidence interval (−47.61, −36.12) supports this contrast.

In Figure 4c, Rq_Walls reveals a positive mean difference (5.20) over Rq_Top, indicating a higher average value for Rq_Walls. The 95% confidence interval (3.20, 7.20) provides an estimated range for this difference. Conversely, Rq_Bottom shows a negative mean difference (−9.59) against Rq_Top, implying a lower average value for Rq_Bottom. The 95% confidence interval (−11.60, −7.59) describes the probable range of this difference. Similarly, Rq_Bottom exhibits a negative mean difference (−14.79) compared to Rq_Walls, indicating a lower mean value for Rq_Bottom. The 95% confidence interval (−16.80, −12.79) substantiates this likely difference.

These findings will guide us to explore the underlying factors contributing to those variations in surface roughness of different sides of FDM printed parts (as discussed in the following Section 4.2).

### 4.2. Mean Interval Plots

**Figure 5:**
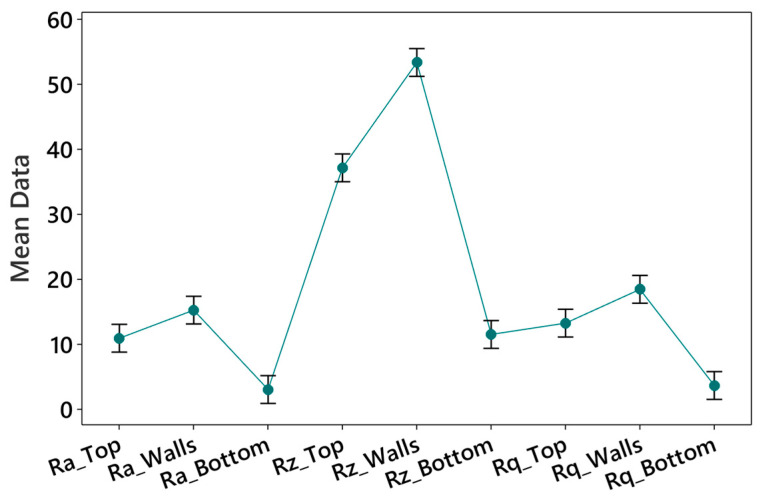
Mean interval plot for surface roughness parameters.

Based on data presented in Table 3, the pooled standard deviation is used to compute the mean intervals of surface roughness parameters, i.e., Ra, Rz, and Rq for the top, wall, and bottom surface of printed parts. It allows for a more accurate assessment of the variability across different sides of printed parts, as illustrated in Figure 5. The mean values of Rz at walls are higher, i.e., 53 μm with an interval of 51 to 55 μm followed by Rz at the top surface having a mean value of 37 μm with an interval of 35 to 39 μm, Rq at walls with mean values of 18.44 μm having an interval of 16.30 to 20.56 μm, and Ra at walls with mean values of 15.24 μm having an interval of 13.10 to 17.28 μm. This suggests that the walls and top surface of the printed parts tend to exhibit higher roughness depths than other sides. The reason may be that the FDM printers build objects layer by layer; as the filament is extruded onto the previous layer, it may slightly deform or flatten against the surface, resulting in variations in the surface texture. These variations can contribute to increased surface roughness, particularly on the walls and top surfaces where the layers are more exposed [21]. Further, after each layer is deposited, it undergoes a cooling and solidification process. The cooling rates can vary across different sides of a printed object, resulting in differential shrinkage and stress [44]. Being more exposed and subject to faster cooling, the walls and top surface may experience more pronounced thermal effects, leading to increased surface roughness [20,21]. Nylon carbon fiber filament materials typically have different thermal expansion coefficients from the base thermoplastic material (such as nylon) and the embedded carbon fibers. As the filament is heated and deposited layer by layer during printing, the thermal expansion mismatch between the two components can result in uneven layer adhesion. This mismatch may lead to delamination or imperfect bonding between the layers, causing surface roughness on the side walls [45]. Further, carbon fibers in nylon carbon fiber filaments introduce anisotropic properties to the printed parts. The orientation and alignment of the carbon fibers can create textured surfaces, leading to higher surface roughness on the side walls. These fibers can protrude or cause surface unevenness, impacting the printed part’s smoothness [46].

On the other hand, it is observed that the mean values of Ra at the bottom (i.e., 3.02 μm with an interval of 0.89 to 5.2 μm) and Rq at the bottom surface (i.e., 3.65 μm with an interval of 1.52 to 5.78 μm) are relatively smaller compared to the surface roughness parameters computed for other sides. This indicates that the bottom surface of the printed parts tends to have a more consistent or less irregular surface compared to the other sides. The bottom surface, which is in contact with the print bed or support structures, may experience more uniform pressure and less disturbance during printing. This can result in more consistent surface roughness with smaller mean intervals. In addition, the heat from the print bed can help improve the first layer’s adhesion and smoothness. The heated bed can melt the plastic slightly, causing it to fuse and create a strong bond with the bed surface [47,48]. However, the support structure’s removal process can sometimes leave minor imperfections, marks, or roughness on the walls and top surface, leading to increased surface roughness. 

### 4.3. Regression Models

Regression models are developed to establish quantitative relationships and provide insights into achieving desired surface characteristics of nylon carbon fiber FDM printed parts. Further, the models will enable quantitative predictions of surface roughness. The regression models developed for each surface roughness parameter (Ra, Rz, and Rq) against different sides (i.e., top, walls, and bottom) are expressed in Equations (8)–(16). It shows that the models are non-linear and quadratic.

(8) $$\begin{array}{ll} Ra_{Walls}= & 119.49+32.57LH+0.96ID+0.003FA-4.29PS-0.013ET+0.311PO-0.0146{ID}^{2}\\ & \;\;\;\;\;\;\;\;\;+0.036{PS}^{2}+0.142LH\times PO-0.001ET\times PO \end{array}$$

(9) $$\begin{array}{ll} Rq_{Walls}= & 151.1+39.73LH+1.141ID-5.391PS-0.0187ET+0.334PO-0.0172{ID}^{2}\\ & \;\;\;\;\;\;\;\;\;+0.0457{PS}^{2}+0.178LH\times PO-0.00027ID\times PO-0.001135ET\times PO \end{array}$$

(10) $$\begin{array}{ll} Rz_{Walls}= & 422.2+113.49LH+3.424ID+0.0112FA-15.16PS-0.049ET+1.065PO\\ & \;\;\;\;\;\;\;\;\;-0.0519{ID}^{2}+0.1286{PS}^{2}+0.5137LH\times PO-0.00378ET\times PO \end{array}$$

(11) $$\begin{array}{ll} Ra_{Top}= & 340.3-48.0LH+2.444ID+0.009FA-11.140PS-0.153ET+0.675PO-0.037{ID}^{2}\\ & \;\;\;\;\;\;\;\;\;+0.094{PS}^{2}+0.499LH\times ET+0.386LH\times PO-0.0024ET\times PO \end{array}$$

(12) $$\begin{array}{ll} Rz_{Top}= & 340.3-48.0LH+2.44ID+0.0088FA-11.140PS-0.153ET+0.675PO-0.037{ID}^{2}\\ & \;\;\;\;\;\;\;\;\;+0.094{PS}^{2}+0.499LH\times ET+0.387LH\times PO-0.0024ET\times PO \end{array}$$

(13) $$\begin{array}{ll} Rq_{Top}= & 340.3-48.0LH+2.44ID+0.0088FA-11.140PS-0.153ET+0.675PO-0.037{ID}^{2}\\ & \;\;\;\;\;\;\;\;\;+0.094{PS}^{2}+0.499LH\times ET+0.39LH\times PO-0.0024ET\times PO \end{array}$$

(14) $$\begin{array}{ll} Ra_{bottom}= & 1.953+11.690LH+0.218NS+0.0012ID-0.00015FA+0.010PS-0.0192BT\\ & \;\;\;\;\;\;\;\;\;-0.0023PO-7.27{LH}^{2}-0.025{NS}^{2}+0.000049{PO}^{2}-0.034LH\times PS\\ & \;\;\;\;\;\;\;\;\;-0.000024PS\times PO \end{array}$$

(15) $$\begin{array}{ll} Rz_{Bottom}= & 14.757+25.586LH+0.484NS+0.004079ID-0.0278PS-0.1402BT-0.01246PO\\ & \;\;\;\;\;\;\;\;\;-0.0851{NS}^{2}-0.00318NS\times PS+0.0057NS\times BT+0.00061PS\times BT\\ & \;\;\;\;\;\;\;\;\;+0.00019BT\times PO \end{array}$$

(16) $$\begin{array}{ll} Rq_{Bottom}= & 4.099+8.0643LH+0.0970NS-0.00091ID+0.0067PS-0.0355BT-0.00511PO\\ & \;\;\;\;\;\;\;\;\;-0.0239{NS}^{2}+0.000605NS\times ID-0.00115NS\times PS+0.00198NS\times BT\\ & \;\;\;\;\;\;\;\;\;+0.000074BT\times PO \end{array}$$

**Figure 6:**
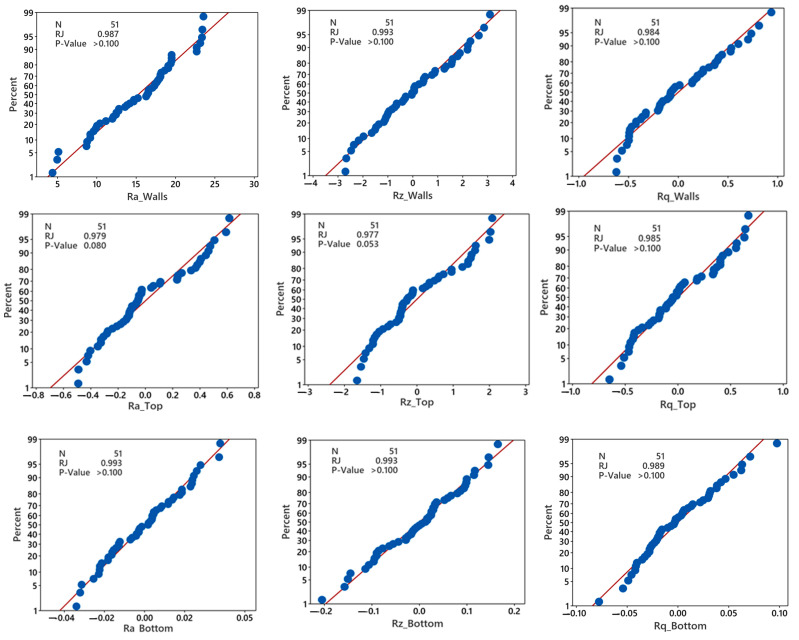
Normality plots for residuals of surface roughness parameters (Ra, Rz, and Rq) on different sides (**top**, **walls**, and **bottom**).

The regression models’ adequacy is evaluated by considering various metrics such as the normality plot for residuals, coefficient of determination (R^2^), adjusted R^2^, predicted R^2^, and lack of fit, as summarized in Figure 6 and Table 5. The normal residual plots depicted in Figure 6 demonstrate that the residuals for each surface parameter closely align with the fitted line, suggesting a normal distribution. Ray Joiner’s (RJ) normality test is conducted with a 95% confidence interval to assess the residuals’ normality further. The test’s null hypothesis assumes that the residuals follow a normal distribution. In contrast, the alternate hypothesis contradicts this assumption by suggesting a *p*-value less than or equal to 0.05. The results of the test indicate that the *p*-value exceeds 0.05. Therefore, the residuals do follow a normal distribution. This reaffirms the developed models’ validity and suitability for making predictions.

The results in Table 5 indicate that the regression models developed for the surface roughness parameters are suitable and effectively represent the experimental data. This conclusion is supported by the high R^2^ values, which approach 100%. The *p*-values obtained from the lack-of-fit test are greater than the significance level of 0.05, indicating that the model fits well with the response data and has no missing terms. The models exhibit satisfactory predictive accuracy, as evidenced by the proximity of the adjusted R^2^ and predicted R^2^ values, with a percentage difference of less than 20%.

### 4.4. Pareto Chart of the Standardized Effects

**Figure 7:**
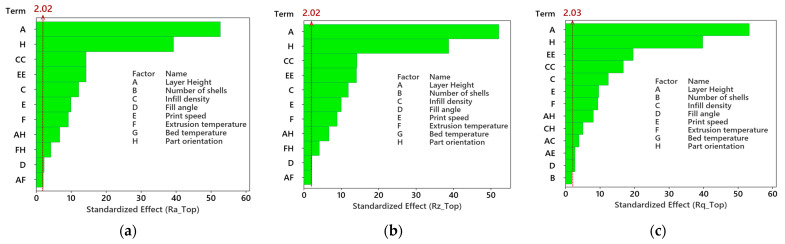

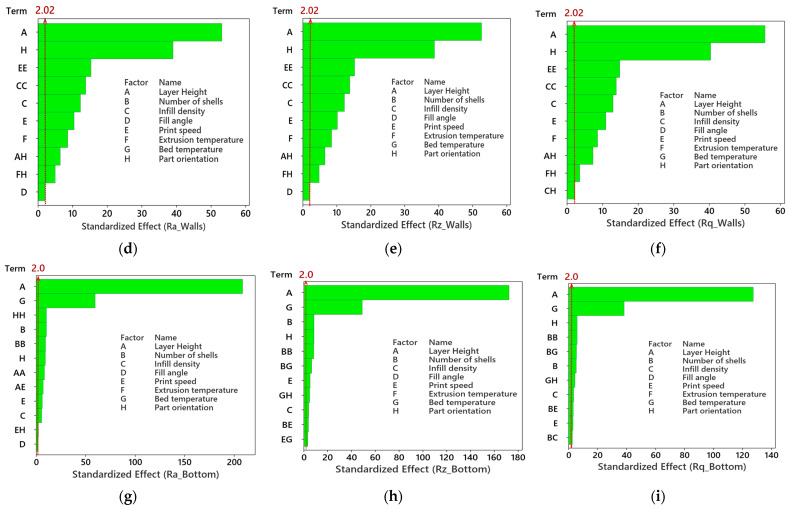
Pareto chart of the standardized effects for surface roughness parameters at different sides; (**a**) average surface roughness at the top surface; (**b**) maximum height of the profile at the top surface; (**c**) root mean square roughness at the top surface; (**d**) average surface roughness of walls; (**e**) maximum height of the profile of walls; (**f**) root mean square roughness of the walls; (**g**) average surface roughness of bottom surface; (**h**) maximum height of the profile of bottom surface; (**i**) root mean square roughness of a bottom surface.

The effect of process parameters on surface roughness parameters was analyzed based on the Pareto chart of the standardized effects. It is an effective tool for identifying the significant factors affecting surface roughness parameters. The results are illustrated in Figure 7a–i. The parameters that crossed the reference line (2 to 2.03) are considered significant at an alpha value of 0.05. Figure 7a–i shows that layer height is the most influential factor that affects the average surface roughness, maximum height of the profile, and root mean square roughness at the top, wall, and bottom surface of nylon carbon fiber printed parts. Part orientation is the second most significant process parameter for Ra, Rz, and Rq at the top and walls (Figure 7a–f). Meanwhile, bed temperature is the second most significant process parameter for Ra, Rz, and Rq at the bottom surface (Figure 7g–i). The other important process parameters commonly found for Ra, Rz, and Rq at the top and walls are infill density and its quadratic term, print speed and its quadratic term, extrusion temperature, the interaction of layer height and part orientation, and a main effect of fill angle. The significant parameters for Ra, Rz, and Rq at the bottom surface are the part orientation, number of shells and their quadratic term, print speed, and infill density.

### 4.5. Surface Plots

Surface plots were generated to study the relationship between the process parameters and the responses.

**Figure 8:**
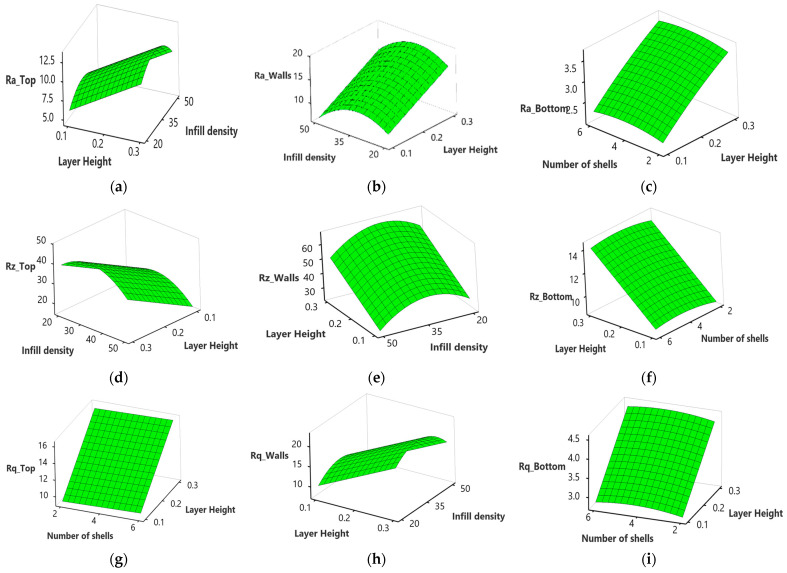
Surface plots for surface roughness; (**a**) effect of layer height and infill density on Ra_Top; (**b**) effect of layer height and infill density on Ra_Walls; (**c**) effect of the number of shells and layer height on Ra_Bottom; (**d**) effect of layer height and infill density on Rz_Top; (**e**) effect of layer height and infill density on Rz_Walls; (**f**) effect of the number of shells and layer height on Rz_Bottom; (**g**) effect of the number of shells and layer height on Rq_Top; (**h**) effect of layer height and infill density on Rq_Walls; (**i**) effect of the number of shells and layer height on Rq_Bottom.

**Figure 9:**
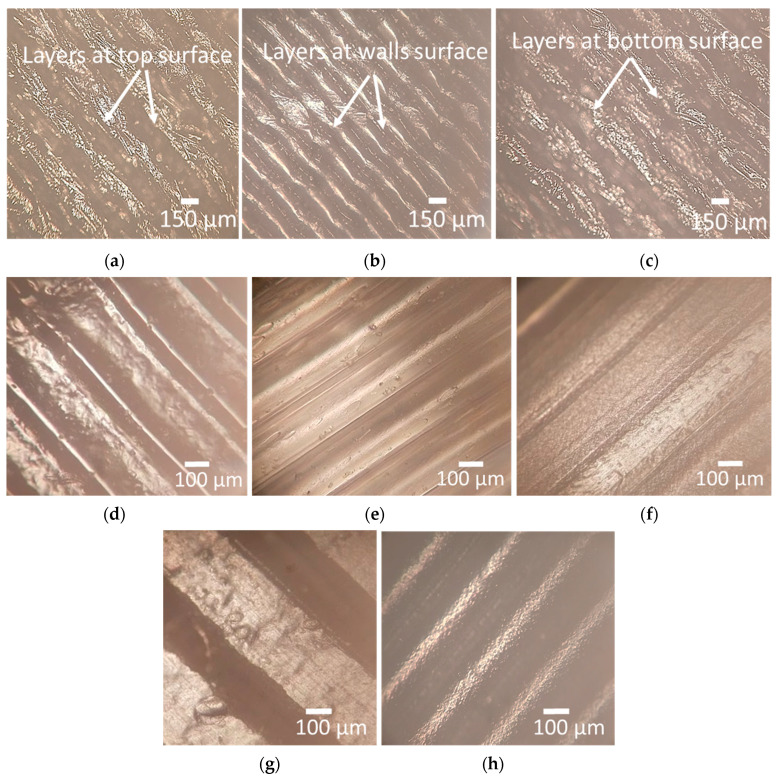
Optical microscopy images; (**a**) top surface of part printed with 0.3 mm layer height; (**b**) wall surface of part printed with 0.3 mm layer height; (**c**) bottom surface of part printed with 0.3 mm layer height; (**d**) top surface of part printed at 20% infill density; (**e**) top surface of part printed at 35% infill density; (**f**) top surface of part printed at 50% infill density; (**g**) bottom surface of part printed with the number of shells of 2; (**h**) bottom surface of part printed with the number of shells of 6.

Figure 8a–i shows that the average surface roughness (Ra), maximum height of the profile (Rz), and root mean square roughness (Rq) of the top, wall, and bottom surface increase with an increase in the layer height. This may be attributed to a decrease in surface contact between the nozzle and the previous layer with an increase in layer height. That results in less efficient flattening and smoothing of the molten filament during deposition, leading to higher surface roughness at the top, wall, and bottom surface, as illustrated in Figure 9a–c obtained using an optical inverted metallurgical microscope (Model No.: M-41X, Lab Testing Technology Shanghai Co., Ltd., Shanghai, China). Further, in larger layer heights, a greater volume of material is deposited in each layer. The deposition of a larger volume of material can lead to more pronounced ridges and valleys on the top surface, contributing to an increase in the maximum height of the profile (Rz) [49]. The surface roughness can be influenced by the carbon fiber reinforcements within the nylon matrix, where a larger layer height may cause variations due to the impact on fiber alignment and distribution within the printed part [50].

There is an increase in Ra, Rz, and Rq at the top and wall surface, with an increase in infill density from 20 to 35%, followed by a decrease from 35 to 50% (Figure 8a,b,d,e,h). At low infill density, there is less material filling the internal regions, resulting in pores and warping during the printing process, leading to increased Ra at the top and walls [51]. However, an increase in infill density minimized the visibility of individual layer lines. As more material is deposited, the surface irregularities caused by the distinct layers may blend, resulting in a perceived decrease in surface roughness at the top and walls [52]. This can be justified further from the microscopic images shown in Figure 9d–f; at a 20% infill density, the layers are more visible than 35% and are blended at 50%.

Ra, Rz, and Rz at the bottom surface increase steadily and then decrease with an increase in the number of shells from 2 to 4 and 4 to 6 (Figure 8c,f,g,i). When more contours or shells are used, the printer creates more layers and fills in more material, resulting in a finer resolution and improved surface quality. The additional contours provide better coverage and fill gaps between the layers, reducing the visibility of the stair-stepping effect, as shown in Figure 9g–h.

**Figure 10:**
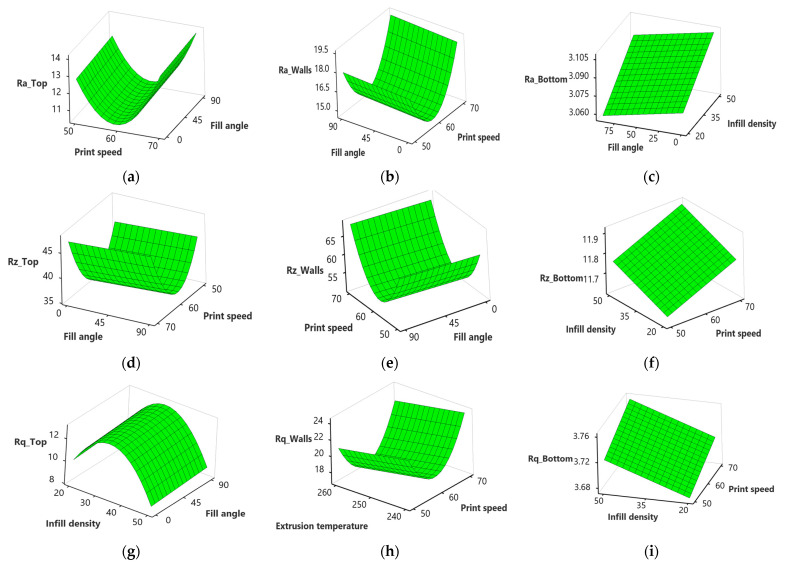
Surface plots for surface roughness; (**a**) effect of print speed and fill angle on Ra_Top; (**b**) effect of fill angle and print speed on Ra_Walls; (**c**) effect of fill angle and infill density on Ra_Bottom; (**d**) effect of fill angle and print speed on Rz_Top; (**e**) effect of print speed and fill angle on Rz_Walls; (**f**) effect of infill density and print speed on Rz_Bottom; (**g**) effect of infill density and fill angle on Rq_Top; (**h**) effect of extrusion temperature and print speed on Rq_Walls; (**i**) effect of the amount of infill density and speed on Rq_Bottom.

Regarding the fill angle, no pronounced effect is observed on Ra, Rz, and Rq at the top and walls with an increase from 0° to 90° (Figure 10a,b,d,e,g). However, Ra at the bottom (Figure 10c) surface decreases with an increase in the fill angle. This is in line with previous studies [53,54] that highlight with the rise in fill angle, the printed layers have a larger contact area between adjacent lines and this allows for better adhesion between layers, resulting in smoother surface finishes.

**Figure 11:**
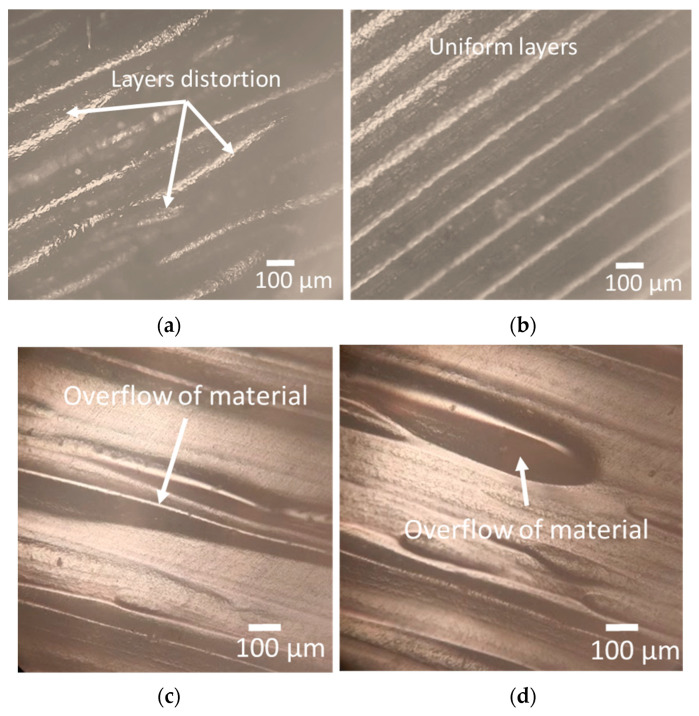
Optical microscopy images; (**a**) layer distortion at a high print speed of 70 mm/s; (**b**) uniform layers at 60 mm/s; (**c**) overflow of material at 50% infill density for part printed at a layer height of 0.1 mm; (**d**) overflow of material at 50% infill density for part printed at a layer height of 0.3 mm.

However, there is a significant decrease in Ra at the top and wall surface with an increase in print speed from 50 mm/s to 60 mm/s, while an increase is observed from 60 to 70 mm/s (Figure 10a,b,d–f,h,i). When the print speed increases, the filament is deposited onto the previous layer more quickly. This faster deposition can lead to better fusion and adhesion between layers, resulting in a smoother surface with reduced surface roughness [55]. However, if the print speed becomes too high, the cooling time for each layer may become insufficient. Inadequate cooling time can cause uneven cooling, resulting in higher residual stresses, warping, or distortions in the printed part. These effects can manifest as increased surface roughness [56]. According to a study [57], high print speed disrupts the continuous flow of material that causes layer distortion, and results in the formation of pores and poor bonding. Figure 11a shows the layer distortion at a high print speed of 70 mm/s, while it was relatively uniform at 60 mm/s, as shown in Figure 11b.

In contrast to the top surface and walls, Ra, Rz, and Rq at the bottom surface increase with an increase in infill density (Figure 10c,f,g,i). This may be attributed to heat transfer and cooling dynamics within the part change. Higher infill densities can result in slower cooling rates and increased shrinkage forces, leading to internal stresses and potential surface roughness, contributing to increased surface roughness at the bottom [58]. The other possible reason is that, as the infill density increases, it becomes more challenging to maintain fine details and intricate features on the bottom surface. Excessive infill density can lead to overfilling, where the material overflows and extends beyond the intended boundaries of the bottom surface. This overflow can create irregularities, protrusions, and a rougher bottom surface, contributing to an increase in surface roughness, as shown in Figure 11c,d. 

Increasing the extrusion temperature from 240 to 260 °C decreases Rq, Ra, and Rz at the top and wall surface (Figure 10h). Increasing the extrusion temperature enhances the melt flow characteristics of the filament. As the filament melts more thoroughly, it has better adhesion and fusion with the previously deposited layers. Improved fusion results in a more cohesive structure and smoother interlayer transitions, reducing surface roughness [59].

**Figure 12:**
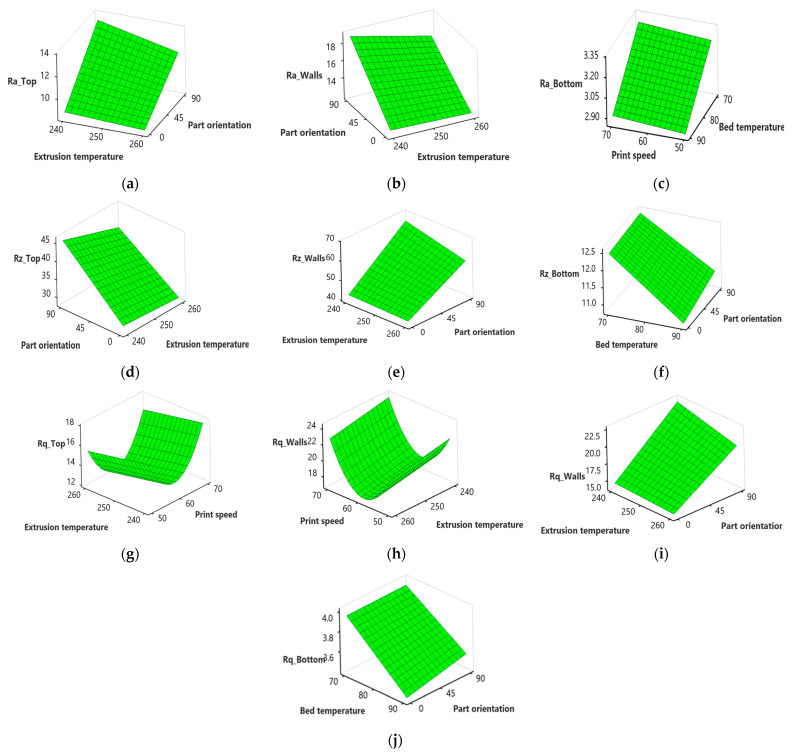
Surface plots for surface roughness; (**a**) effect of extrusion temperature and part orientation on Ra_Top; (**b**) effect of extrusion temperature and part orientation on Ra_Walls; (**c**) effect of bed temperature and print speed on Ra_Bottom; (**d**) effect of extrusion temperature and part orientation on Rz_Top; (**e**) effect of extrusion temperature and part orientation on Rz_Walls; (**f**) effect of bed temperature and part orientation on Rz_Bottom; (**g**) effect of extrusion temperature and print speed on Rq_Top; (**h**) effect of extrusion temperature and print speed on Rq_Walls; (**i**) effect of extrusion temperature and part orientation on Rq_Walls; (**j**) effect of bed temperature and part orientation on Rq_Bottom.

**Figure 13:**
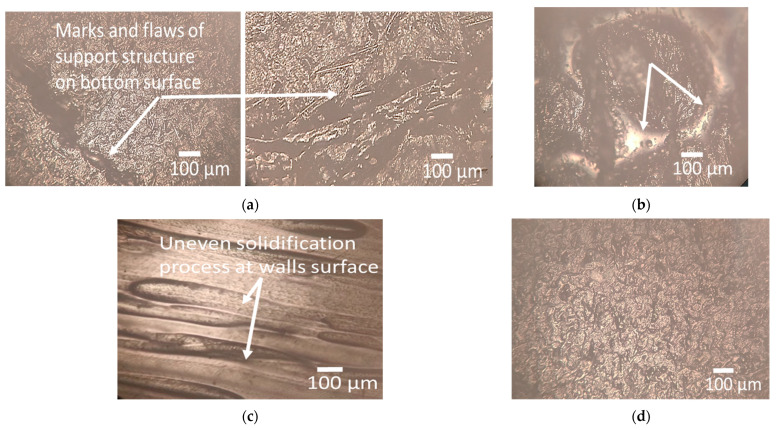
Optical microscopy images; (**a**) marks and flaws of support structure on the bottom surface; (**b**) uneven solidification process at the top surface; (**c**) uneven solidification process at the wall surface; (**d**) smooth bottom surface.

On the other hand, there is a drastic increase in Ra, Rz, and Rq at the top, wall, and bottom surface with an increase in part orientation from 0° to 90° (Figure 12a,b,d–f,i,j). This may be attributed to the support structure that leaves behind small imperfections, marks, or roughness on the bottom surface, leading to increased surface roughness, as evident from a microscopic structural analysis as illustrated in Figure 13a. The top surface of a printed part is exposed to the air, which can result in faster cooling compared to other surfaces in contact with the print bed or support structures. Rapid filament cooling can cause uneven solidification and increase surface roughness, as shown in Figure 13b. When printing in vertical orientations, the reduced contact with the print bed restricts heat dissipation, leading to less effective cooling and higher surface roughness [60,61]. When printing vertically (90° orientation), the layers are stacked on top of each other, and the deposition direction is perpendicular to the top surface. This orientation can result in step-like features, layer mismatches, and reduced surface quality, increasing Rz [62,63].

However, there is a sharp decrease with an increase in bed temperature from 70 to 90 °C (Figure 12c,f,j). Increasing the bed temperature improves adhesion between the printed part and the build platform. Adequate bed adhesion ensures that the bottom layers of the print remain firmly attached during the printing process, reducing the chances of warping or shifting. This improved adhesion results in a smoother and more uniform bottom surface with reduced surface roughness, as evident from Figure 13d. According to studies [64,65], higher bed temperatures can help to mitigate part distortions, particularly at the bottom surface. When the bed temperature increases, the layers in contact with the build platform experience less differential cooling and shrinkage. This reduces the likelihood of warping, curling, or other deformations contributing to surface roughness.

By analyzing the trends, it is evident that the choice of printing parameters significantly influences surface roughness. For optimal results, it is recommended to carefully consider the desired surface quality requirements for each specific surface. Adjusting parameters such as layer height, infill density, print speed, and extrusion temperature can help achieve the desired surface roughness characteristics. Furthermore, the findings highlight the importance of considering different surfaces, such as the top, walls, and bottom surfaces, as their responses to printing parameters may vary. This knowledge can guide manufacturers and 3D printing enthusiasts in making informed decisions when selecting printing parameters to achieve the desired surface roughness for their specific applications. Further research and experimentation may be required to fully explore the interactions between different printing parameters and their impact on surface roughness. Nevertheless, the presented trends serve as a valuable starting point for optimizing the printing process and enhancing the surface quality of 3D printed objects.

### 4.6. Multi-Response Optimization

The desirability function (DF) of individual responses (in Table 3) is computed using Equation (1), as shown in Table 6. To calculate the weights of responses, determine the entropy for each DF using Equations (3)–(5). Determine the redundancy of entropy using Equation (6) and compute the weights based on Equation (7). The results are summarized in Table 7. It shows that the highest weight is attributed to ${Rq\;}_{B}$ (0.132) followed by ${Rz\;}_{B}$ (0.126), ${Ra\;}_{B}$ (0.124), and ${Rq\;}_{T}$ (0.106), while the least weight is assigned to ${Rz\;}_{w}$ (0.132). Finally, the weighted composite desirability function (WCDF) is determined using Equation (2) and is tabulated in Table 6. Finally, to determine the optimal levels, the average values of the Weighted Cumulative Distribution Function (WCDF) are calculated for each parameter across different levels, as presented in Table 8. The highest average WCDF value among the three levels for each parameter indicates the optimal level for that specific parameter. The optimal level for the layer height and number of shells is the low level (−1), showing the highest WCDF averages of 0.706 and 0.467, respectively, having values of 0.1 mm and two shells. Similarly, for infill density, the highest WCDF average obtained is 0.487 at the high level (1), corresponding to a 50% infill density. For the infill angle, the optimal level is the low level (−1), yielding a WCDF average of 0.461, denoting a 0° fill angle. For print speed, the medium level (0) prevails with a WCDF average peak of 0.532, equating to 60 mm/s. Notably, nozzle temperature’s high level (1) showcases the highest WCDF average (0.466) at 260 °C, while bed temperature’s medium level (0) excels with a 0.495 WCDF average at 80 °C. Lastly, print orientation’s optimal level is the low level (−1), capturing the highest WCDF average of 0.544 at a 0° orientation. The optimized values obtained for the specimens printed at optimal levels are the ${(Ra,\;Rz,\;Rq)\;}_{walls}\;$ of (3.95, 13.83, 4.74) µm, for ${(Ra,\;Rz,\;Rq)\;}_{Top}$, they are (2.82, 10.03, 3.38) µm, and for ${(Ra,\;Rz,\;Rq)\;}_{Bottom}$, they are (1.92, 6.72, 2.31) µm.

## 5. Conclusions

Fused deposition modeling (FDM) is a cost-effective method for creating engineering components through additive manufacturing. However, controlling surface roughness poses challenges for functional part fabrication due to numerous process parameters that need to be considered during printing. So, the present study performed optimization and detailed investigation and comprehended the impact of FDM process parameters on surface roughness parameters (Ra, Rq, and Rz) across different sides (bottom, top, and walls) of nylon carbon fiber printed components. Based on experimental results and statistical investigation, the following conclusions are drawn:

- The surface roughness (Ra, Rq, and Rz) is higher for walls than the top, while the bottom has the lowest roughness as per results obtained using an analysis of variance.
- The Pareto chart analysis revealed that layer height is the most influential factor affecting surface roughness parameters, followed by part orientation and bed temperature. Other significant factors include the infill density, print speed, extrusion temperature, fill angle, number of shells, and their respective quadratic terms, affecting surface roughness at different parts of nylon carbon fiber printed parts.
- Higher layer heights increase roughness, while more infill density and shells improve surface quality. Adjusting fill angle and print speed impacts roughness on different parts—the bottom or top. Part orientation, especially from 0° to 90°, increases roughness due to overhangs. Bed temperature (70–90 °C) improves bottom surface smoothness via enhanced adhesion and minimized distortions.
- The optimized level obtained based on an integrated approach of composite desirability function (CDF) and entropy is the layer height of 0.1 mm, number of shells of two, infill density of 50%, print speed of 60 mm/s, extrusion temperature of 260 °C, bed temperature of 80 °C, and print orientation of 0°. The optimized values obtained for ${(Ra,\;Rz,\;Rq)\;}_{walls}\;$ are (3.95, 13.83, 4.74) µm, for ${(Ra,\;Rz,\;Rq)\;}_{Top}$, they are (2.82, 10.03, 3.38) µm, and for ${(Ra,\;Rz,\;Rq)\;}_{Bottom}$, they are (1.92, 6.72, 2.31) µm.

### Limitations and Future Prospects

The implications of the present study can be extended beyond nylon carbon fiber composites, with principles applicable to diverse FDM materials. Although optimal parameter values may differ per material, the systematic approach to a parameter analysis and optimization remains valid. However, an atomic force microscopy (AFM) and scanning electron microscopy (SEM) analysis will provide better understanding of roughness parameters that will be used in future studies. As additive manufacturing evolves, surface quality’s importance for function and aesthetics persists. Our study offers a foundational understanding, empowering practitioners to enhance surface quality across materials like PLA, ABS, PETG, and more, fostering superior 3D printing outcomes. 

Future research can further explore additional factors and techniques to enhance surface quality in additive manufacturing processes.

## Figures and Tables

**Table 1:** Printing parameters with three levels.

Printing Parameters	Units	Symbol	Levels		
			−1	0	1
Layer height	mm	LH	0.1	0.2	0.3
Printing speed	mm/s	PS	50	60	70
Number of shells	-	NS	2	4	6
Infill density	%	ID	20	35	50
Part orientation	°	PO	0	45	90
Fill angle	°	FA	0	45	90
Extrusion temperature	°C	ET	240	250	260
Bed temperature	°C	BT	70	80	90

**Table 2:** Experimental runs based on definitive screening design.

Exp. No	LH	NS	ID	FA	PS	NT	BT	PO	Exp. No	LH	NS	ID	FA	PS	NT	BT	PO
1	0.2	6	50	90	70	260	90	90	27	0.2	2	20	0	50	240	70	0
2	0.1	2	50	90	70	250	70	0	28	0.1	4	20	90	50	260	90	0
3	0.3	6	20	90	70	240	80	0	29	0.3	6	50	45	50	260	70	0
4	0.1	6	35	90	50	240	70	90	30	0.1	2	50	90	70	250	70	0
5	0.3	6	50	45	50	260	70	0	31	0.2	4	35	45	60	250	80	45
6	0.3	6	20	0	50	250	90	90	32	0.2	2	20	0	50	240	70	0
7	0.3	6	20	90	70	240	80	0	33	0.1	6	50	0	60	240	90	0
8	0.1	2	50	0	50	260	80	90	34	0.3	2	50	90	50	240	90	45
9	0.3	2	20	90	60	260	70	90	35	0.3	6	50	45	50	260	70	0
10	0.3	6	20	0	50	250	90	90	36	0.3	4	50	0	70	240	70	90
11	0.1	2	50	0	50	260	80	90	37	0.3	4	50	0	70	240	70	90
12	0.1	6	50	0	60	240	90	0	38	0.2	6	50	90	70	260	90	90
13	0.1	2	20	45	70	240	90	90	39	0.3	2	50	90	50	240	90	45
14	0.1	6	20	0	70	260	70	45	40	0.1	4	20	90	50	260	90	0
15	0.1	6	20	0	70	260	70	45	41	0.2	6	50	90	70	260	90	90
16	0.1	2	50	90	70	250	70	0	42	0.3	2	35	0	70	260	90	0
17	0.1	6	50	0	60	240	90	0	43	0.1	6	35	90	50	240	70	90
18	0.3	2	35	0	70	260	90	0	44	0.2	2	20	0	50	240	70	0
19	0.1	6	20	0	70	260	70	45	45	0.3	2	50	90	50	240	90	45
20	0.1	2	50	0	50	260	80	90	46	0.1	2	20	45	70	240	90	90
21	0.1	2	20	45	70	240	90	90	47	0.2	4	35	45	60	250	80	45
22	0.1	6	35	90	50	240	70	90	48	0.3	6	20	0	50	250	90	90
23	0.2	4	35	45	60	250	80	45	49	0.3	2	20	90	60	260	70	90
24	0.1	4	20	90	50	260	90	0	50	0.3	4	50	0	70	240	70	90
25	0.3	2	35	0	70	260	90	0	51	0.3	2	20	90	60	260	70	90
26	0.3	6	20	90	70	240	80	0									

**Table 3:** Measured surface parameters based on definitive screening design.

Exp. No	Wall			Top			Bottom		
	Ra	Rz	Rq	Ra	Rz	Rq	Ra	Rz	Rq
1	17.05	59.68	20.29	12.12	41.21	14.67	2.95	11.21	3.54
2	9.17	32.19	11.10	6.55	22.27	7.86	2.55	9.69	3.14
3	17.42	61.32	21.08	12.44	42.30	15.05	3.65	13.87	4.38
4	16.55	58.42	20.03	12.45	42.33	15.06	2.52	9.60	3.02
5	13.86	48.51	16.77	9.92	33.93	12.20	3.88	14.74	4.77
6	22.67	79.34	28.11	16.19	55.05	19.59	3.58	13.60	4.30
7	18.07	63.25	21.86	12.91	43.89	15.62	3.62	13.79	4.34
8	10.39	36.37	12.57	7.42	25.23	8.76	2.32	8.82	2.78
9	19.39	67.87	23.46	13.85	47.37	16.76	3.85	14.63	4.74
10	23.41	81.94	28.33	16.75	56.95	20.27	3.55	13.56	4.26
11	10.02	34.97	12.52	7.06	24.00	8.54	2.29	8.70	2.75
12	4.97	17.39	6.01	3.55	12.07	4.22	2.16	8.21	2.64
13	16.53	57.86	20.00	11.81	40.15	14.29	2.15	8.17	2.58
14	12.42	43.47	15.03	8.87	29.98	10.73	2.49	9.54	2.99
15	12.75	44.63	15.43	9.11	30.97	11.11	2.51	9.54	3.01
16	9.87	34.35	12.44	7.05	23.97	8.53	2.51	9.54	3.09
17	5.12	17.92	6.20	4.06	13.80	4.95	2.17	8.27	2.60
18	19.50	68.25	23.59	13.93	48.06	16.86	3.42	13.00	4.10
19	11.98	41.93	14.50	8.56	29.10	10.53	2.53	9.61	3.04
20	11.14	38.99	13.15	7.96	27.06	9.63	2.34	8.94	2.88
21	16.39	56.71	19.83	11.72	39.85	14.18	2.17	8.25	2.60
22	18.08	63.28	21.88	13.13	45.30	15.89	2.58	9.80	3.10
23	14.70	51.45	17.49	10.52	35.77	12.73	3.08	11.80	3.79
24	8.67	30.34	10.49	6.19	21.05	7.49	2.12	8.06	2.54
25	19.51	68.29	23.61	13.95	47.43	16.88	3.43	13.08	4.06
26	17.29	60.52	20.92	12.35	41.99	14.94	3.68	13.98	4.42
27	12.80	44.80	15.49	9.14	31.08	11.06	3.17	11.76	3.80
28	9.16	32.06	11.08	6.54	22.24	7.91	2.14	8.13	2.57
29	14.69	51.41	17.77	10.49	35.67	12.69	3.92	14.90	4.70
30	8.75	30.63	10.59	6.25	21.25	7.56	2.57	9.77	3.08
31	15.22	53.27	18.42	10.87	36.96	13.15	3.06	11.63	3.67
32	12.49	43.72	15.11	8.92	30.33	10.79	3.15	12.13	3.78
33	4.37	15.29	5.29	3.12	10.61	3.78	2.16	8.21	2.59
34	19.07	66.75	23.07	13.62	46.31	16.48	3.38	12.84	4.06
35	13.59	47.56	16.44	9.71	33.01	11.75	3.90	14.82	4.68
36	23.14	80.99	28.00	16.53	56.37	20.00	4.01	15.24	4.81
37	23.35	81.73	28.25	16.68	56.71	20.18	3.97	15.09	4.76
38	17.60	61.60	21.30	12.57	43.12	15.21	3.01	11.44	3.61
39	17.95	61.93	21.72	12.82	43.59	15.51	3.36	12.77	4.03
40	9.60	33.60	11.62	6.86	23.32	8.30	2.13	8.09	2.56
41	16.67	58.35	20.17	11.91	40.49	14.41	2.97	11.29	3.56
42	19.50	68.25	23.59	13.93	47.36	16.86	3.45	13.13	4.14
43	18.18	64.18	20.91	13.10	43.75	16.38	2.53	9.61	3.04
44	12.11	42.38	14.65	8.65	29.41	10.47	3.18	12.08	3.85
45	17.75	62.13	21.48	12.68	43.11	15.85	3.40	12.92	4.08
46	16.27	56.95	19.69	11.62	39.51	14.06	2.18	8.28	2.62
47	14.20	49.70	17.32	10.14	34.68	12.27	3.10	11.78	3.75
48	22.67	80.03	27.43	16.19	55.05	19.75	3.57	13.67	4.32
49	18.61	65.51	22.52	13.31	45.25	16.11	3.83	14.67	4.60
50	23.55	82.90	28.50	16.82	57.52	20.35	4.02	15.28	4.82
51	19.08	66.78	23.28	13.66	46.58	16.94	3.84	14.71	4.72

**Table 4:** Analysis of Variance (ANOVA).

Source	F-Value	*p*-Value
Ra (Walls, Top, and Bottom)	157.81	<0.001
Rz (Walls, Top, and Bottom)	151.28	<0.001
Rq (Walls, Top, and Bottom)	157.6	<0.001

**Table 5:** Summary of regression model adequacy.

Responses	R^2^ (%)	Adjusted R^2^ (%)	Predicted R^2^ (%)	Lack of Fit Based on *p*-Value
Ra_Walls	99.26	99.07	98.8	0.637
Rz_Walls	99.24	99.06	98.78	0.51
Rq_Walls	99.53	99.42	99.24	0.802
Ra_Top	99.28	99.07	98.77	0.43
Rz_Top	99.26	99.05	98.74	0.539
Rq_Top	99.33	99.09	98.71	0.898
Ra_Bottom	99.92	99.89	99.85	0.816
Rz_Bottom	99.88	99.85	99.79	0.271
Rq_Bottom	99.78	99.72	99.64	0.575

**Table 6:** Desirability function (DF) and weighted composite desirability function (WCDF).

Exp. No	Wall (DF)			Top (DF)			Bottom (DF)			WCDF
	Ra	Rz	Rq	Ra	Rz	Rq	Ra	Rz	Rq	
1	0.339	0.343	0.354	0.343	0.348	0.343	0.563	0.564	0.561	0.416
2	0.75	0.75	0.75	0.75	0.751	0.754	0.774	0.774	0.737	0.755
3	0.32	0.319	0.32	0.32	0.324	0.32	0.195	0.195	0.193	0.265
4	0.365	0.362	0.365	0.319	0.324	0.319	0.789	0.787	0.789	0.470
5	0.505	0.509	0.505	0.504	0.503	0.492	0.074	0.075	0.022	0.206
6	0.046	0.053	0.017	0.046	0.053	0.046	0.232	0.233	0.228	0.079
7	0.286	0.291	0.286	0.285	0.291	0.285	0.211	0.206	0.211	0.254
8	0.686	0.688	0.686	0.686	0.688	0.699	0.895	0.895	0.895	0.761
9	0.217	0.222	0.217	0.217	0.216	0.217	0.089	0.09	0.035	0.137
10	0.007	0.014	0.007	0.005	0.012	0.005	0.247	0.238	0.246	0.029
11	0.705	0.709	0.688	0.712	0.715	0.713	0.911	0.911	0.908	0.779
12	0.969	0.969	0.969	0.969	0.969	0.973	0.979	0.979	0.956	0.970
13	0.366	0.37	0.366	0.366	0.37	0.366	0.984	0.985	0.982	0.535
14	0.58	0.583	0.58	0.58	0.587	0.581	0.805	0.795	0.803	0.657
15	0.563	0.566	0.563	0.563	0.566	0.558	0.795	0.795	0.794	0.642
16	0.713	0.718	0.692	0.713	0.715	0.713	0.795	0.795	0.759	0.737
17	0.961	0.961	0.961	0.931	0.932	0.929	0.974	0.971	0.974	0.956
18	0.211	0.217	0.212	0.211	0.202	0.211	0.316	0.316	0.316	0.246
19	0.603	0.606	0.603	0.603	0.606	0.593	0.784	0.785	0.781	0.666
20	0.647	0.649	0.661	0.647	0.649	0.647	0.884	0.878	0.851	0.727
21	0.373	0.387	0.374	0.372	0.377	0.372	0.974	0.974	0.974	0.541
22	0.285	0.29	0.285	0.269	0.26	0.269	0.758	0.759	0.754	0.406
23	0.461	0.465	0.474	0.46	0.464	0.46	0.495	0.482	0.452	0.468
24	0.776	0.777	0.776	0.776	0.777	0.776	1	1	1	0.855
25	0.211	0.216	0.211	0.209	0.215	0.209	0.311	0.305	0.333	0.247
26	0.326	0.331	0.327	0.326	0.331	0.326	0.179	0.18	0.175	0.260
27	0.56	0.564	0.561	0.561	0.564	0.561	0.447	0.488	0.447	0.520
28	0.75	0.752	0.751	0.75	0.752	0.751	0.989	0.99	0.987	0.834
29	0.462	0.466	0.462	0.462	0.466	0.462	0.053	0.053	0.053	0.202
30	0.772	0.773	0.772	0.772	0.773	0.772	0.763	0.763	0.763	0.769
31	0.434	0.438	0.434	0.434	0.438	0.435	0.505	0.506	0.504	0.461
32	0.577	0.58	0.577	0.577	0.58	0.577	0.458	0.436	0.456	0.525
33	1	1	1	1	1	1	0.979	0.979	0.978	0.992
34	0.234	0.239	0.234	0.234	0.239	0.234	0.337	0.338	0.333	0.270
35	0.519	0.523	0.52	0.519	0.522	0.519	0.063	0.064	0.061	0.232
36	0.021	0.028	0.022	0.021	0.025	0.021	0.005	0.006	0.004	0.013
37	0.01	0.017	0.011	0.01	0.017	0.01	0.026	0.026	0.026	0.016
38	0.31	0.315	0.31	0.31	0.307	0.31	0.532	0.532	0.531	0.381
39	0.292	0.31	0.292	0.292	0.297	0.292	0.347	0.348	0.346	0.314
40	0.727	0.729	0.727	0.727	0.729	0.727	0.995	0.996	0.991	0.820
41	0.359	0.363	0.359	0.358	0.363	0.358	0.553	0.553	0.553	0.424
42	0.211	0.217	0.212	0.211	0.217	0.211	0.3	0.298	0.298	0.242
43	0.28	0.277	0.327	0.272	0.294	0.24	0.784	0.785	0.781	0.415
44	0.596	0.599	0.597	0.596	0.599	0.596	0.442	0.443	0.425	0.530
45	0.302	0.307	0.302	0.302	0.307	0.272	0.326	0.327	0.325	0.309
46	0.38	0.384	0.38	0.38	0.384	0.38	0.968	0.97	0.965	0.544
47	0.487	0.491	0.482	0.488	0.487	0.488	0.484	0.485	0.469	0.484
48	0.046	0.042	0.046	0.046	0.053	0.036	0.237	0.223	0.219	0.083
49	0.258	0.257	0.258	0.256	0.262	0.256	0.1	0.084	0.096	0.175
50	0	0	0	0	0	0	0	0	0	0.000
51	0.233	0.238	0.225	0.231	0.233	0.206	0.095	0.079	0.044	0.144

**Table 7:** Weights of responses based on entropy.

Responses	(${Ra}_{w}$)	(${Rz}_{w}$)	(${Rq}_{w}$)	(${Ra}_{T}$)	(${Rz}_{T}$)	(${Rq}_{T}$)	(${Ra}_{B}$)	(${Rz}_{B}$)	(${Rq}_{B}$)
$e_{j}$	0.949	0.951	0.948	0.949	0.950	0.947	0.939	0.938	0.935
$d_{j}$	0.051	0.049	0.052	0.051	0.050	0.053	0.061	0.062	0.065
$w_{j}$	0.103	0.100	0.104	0.104	0.101	0.106	0.124	0.126	0.132

**Table 8:** Optimal levels based on average values of weighted composite desirability function (WCDF).

Process Parameters	Levels			Optimal Levels
	−1	0	1	
Layer height	0.706	0.468	0.177	−1 (0.1 mm)
Number of shells	0.467	0.439	0.429	−1 (2)
Infill density	0.433	0.382	0.487	1 (50%)
Fill angle	0.461	0.408	0.448	−1 (0°)
Print speed	0.446	0.532	0.410	0 (60 mm/s)
Nozzle temperature	0.434	0.429	0.466	1 (260 °C)
Bed temperature	0.391	0.495	0.480	0 (80 °C)
Print orientation	0.544	0.475	0.337	−1 (0°)

## Data Availability

All the data are available in the paper.

## List of Abbreviations

AM	Additive manufacturing
FDM	Fused deposition modeling
Ra	Average surface roughness
Rq	Root mean square surface roughness
Rz	Maximum height of the profile
DSD	Definitive screening design
ANOVA	Analysis of variance
ABS	Acrylonitrile butadiene styrene
PLA	Poly-lactic acid
PA	Polyamide
PETG	Polyethylene terephthalate glycol
PEEK	Polyetheretherketone
CDF	Composite desirability function
WCDF	Weighted composite desirability function
